# Behavioral Crisis and First Response: Qualitative Interviews with Chicago Stakeholders

**DOI:** 10.1007/s10597-022-00990-2

**Published:** 2022-06-25

**Authors:** Conor H. Murray, Juan L. Contreras, Caroline H. Kelly, Deborah K. Padgett, Harold A. Pollack

**Affiliations:** 1 Department of Psychiatry and Behavioral Neuroscience, University of Chicago, 5841 S Maryland Ave, Chicago, IL 60637 USA; 2 Urban Health Lab, University of Chicago, Chicago, USA; 3 Crown Family School of Social Work Policy and Practice, University of Chicago, 969 E 60th St, Chicago, IL 60637 USA; 4 Silver School of Social Work, New York University, 1 Washington Square N, New York, NY 10003 USA; 5 Department of Public Health Sciences, University of Chicago, 5841 S Maryland Ave, Chicago, IL 60637 USA

**Keywords:** Behavioral crisis, First-response, 911, CIT, Psychosis, SUD

## Abstract

Improving interactions between first responders and individuals experiencing behavioral crisis is a critical public health challenge. To gain insight into these interactions, key informant qualitative interviews were conducted with 25 Chicago stakeholders. Stakeholders included directors and staff of community organizations and shelters that frequently engage first responders. Interviews included granular depictions related to the expectations and outcomes of 911 behavioral crisis calls, and noted areas requiring improved response. Stakeholders called 911 an average of 2 to 3 times per month, most often for assistance related to involuntary hospitalization. Engagements with first responders included unnecessary escalation or coercive tactics, or conversely, refusal of service. While stakeholders lauded the value of police trained through the city’s Crisis Intervention Team program, they emphasized the need for additional response strategies that reduce the role of armed police, and underscored the need for broader social and behavioral health services for individuals at-risk of such crises.

## Introduction

Encounters between emergency first-responders and individuals experiencing behavioral crisis introduce risks of escalation and violence, posing a serious public health and safety challenge (Cordner, 2006; Deane et al., 1999; Teplin et al., 1992). Individuals with mental illness are three times more likely to interact with police (Hoch et al., 2009), and individuals with serious mental illness are 16 times more likely to be killed by police (Fuller et al., 2015). Encounters between police and individuals with mental illness, and the interaction between the criminal justice and health care systems in the United States has been a major topic of interest following policy shifts in the standard practice of care for individualswith mental illness from psychiatric hospitals to community treatment settings, coinciding with in an overrepresentation of mentally ill individuals in the criminal justice system (Bonfine et al., 2020; Teplin & Teplin, 1983). Recently, researchers have advocated for next-generation behavioral health and criminal justice interventions and have discussed possible approaches for successful outcomes in crisis response (Epperson et al., 2014; Watson et al., 2021).

In Chicago, our study site, efforts are underway to train and deploy police officers to recognize and de-escalate behavioral crises, most prominently through crisis intervention teams (CIT). Our prior research has explored the effectiveness of Crisis Intervention Team (CIT) training and other approaches to immediately manage first response to behavioral health crises (Compton et al., 2014; Wood et al., 2021). Although rigorous evaluation is limited, existing studies suggest that such efforts improve officers’ attitudes and self-efficacy in response to behavioral crises, and that such training likely improves safety of the specific encounters (Compton et al., 2010; Watson & Compton, 2019; Watson et al., 2017, 2021). Our work, and the work of others, has linked clinical and criminal justice data systems to determine rates of arrests and the risk of repeat arrest among individuals with mental illness to identify opportunities for non-violent interventions (Compton 2014; Livingston & Livingston, 2016; Magee et al., 2021; Tentner et al., 2019; Wood et al., 2021).

The perspectives of behavioral health and social-service providers who may call upon first responders receive less systematic attention. The experiences of these providers may be valuable for policy makers in establishing more efficient and effective interactions between first responders and individuals with mental illness. In this study, our objective was to determine when and why providers seek assistance from first responders, how the response unfolds, what the outcomes of the response entail, and whether providers have pain points or recommendations for response improvement. We conducted 25 key informant qualitative interviews with staff members from shelters, group-homes, and social-service organizations—locations deemed high-risk for emergency encounters during behavioral health crises.

## Methods

### Study Setting and Recruitment

Twenty-five mental health and social service professionals were recruited from Chicago homeless shelters, group homes, mental health, and substance use disorder (SUD) treatment facilities that were selected based on our prior work (Tentner et al., 2019), as locations that made frequent contacts with crisis responders. Recruitment occurred through email, phone, and snowball sampling. To ensure that the identities of the providers are protected, we do not provide identifiable information related to these agencies, including agency names, specific job titles, or specific services a particular agency provides. As a whole, the individuals that were recruited for interviews represent agencies that serve people with serious mental illnesses who may have co-occurring substance use disorders. Each respondent electronically consented to study procedures via REDCap, a secure web application for building and managing online surveys and databases. Interviews took place during the year 2020 and respondents were asked to reflect on responses through a pre-COVID-19 lens.

### Procedure and Protocol

All procedures and protocols were approved by our Institutional Review Board. Following recruitment and consent, respondents completed a background demographic questionnaire via REDCap. Thereafter, one of three semi-structured interview guides were used during a confidential, approximately-45-min Zoom video call by one of three study team members. The three guides were used for three specific types of agencies: one general guide for shelters and group homes, and two guides specific to two major agencies in the city of Chicago involved in community services. All three interview guides contained similar questions and probes to address the study aims, including respondents’ experiences with behavioral health crises, decisions to call—or not to call—911 when behavioral crises occur, role of CIT trained officers, existing training and protocols to reduce the frequency and severity of 911 calls, specific barriers to effective response, and broader policies and practices desired to meet the needs of individuals vulnerable to behavioral crises. However, only the general interview guide probed respondents on a quantitative measure of 911 call frequency per month. Respondents were compensated with a $20 e-gift card following interview completion.

### Data Analysis

Interviews were transcribed by Rev.com. Transcriptions were imported into the NVivo Version 12 software package. Data were analyzed using principles of grounded theory, including theme identification (Padgett, 2016). Grounded theory was selected (Teherani et al., 2015) as a means of comparing across diverse stakeholder groups (homeless shelters, group homes, community and addiction services), toward our goal of identifying themes that capture key features of the life cycle of the behavioral health crisis response, from issues surrounding crisis encounters to possible solutions that reduce their frequency and severity. To this end, three analysts developed codes based on interview guides and sensitizing concepts; codes were not selected*a priori*. NVivo was used to apply codes and to tag related text segments. Analysts worked independently and then in tandem to consensually develop a codebook. Each interview was independently coded twice to reduce bias and enhance intercoder agreement (Wood et al., 2021); however no inter rater reliability was calculated. Emergent themes were identified after repeated independent reading and memoing intended to arrive at a consensus.

## Results

Most respondents were female (68%), white (80%), and held master’s degrees (64%). Most had 1–5 years of experience in their current positions, either as frontline service providers (72%) or as managerial directors (28%). Our findings below document emergent themes that capture key features of the life cycle of the behavioral health crisis response, from issues surrounding crisis encounters to possible solutions that reduce their frequency and severity.

### Crisis Conceptualization

When respondents described their experiences with behavioral crises, many often described acute episodes triggered by symptoms associated with comorbid psychosis and SUD, as exemplified by quotes throughout this section. However, this acute conceptualization of crisis was also challenged. Provider 10 stated, “I have to look at all of them as always being in crisis because if I didn’t have a home, a bed to sleep in, and know where my next meal is coming from, I would be in crisis.”

#### Comorbidity and Dual-diagnoses

Most respondents underscored the importance of co-occurring SUD and serious mental illness (SMI) diagnoses. Dual-diagnosis was described as a particular catalyst for behavioral dysregulation that can pose safety concerns to other clients, staff, to first responders, and to the client themselves. Director 3 related: “…folks who are using substances, primarily alcohol and stimulants, we see an uptick in aggressive behavior. The aggression can come out from screaming and yelling to throwing things at staff.”

#### Mental Illness and Safety Concerns

Director 6 shared an example in which mental illness severity threatened clients’ own safety, noting: “Significant depression might lead to suicidal thoughts or self-harm.” Provider 18 added: “[A] member who’s been particularly more depressed lately… potentially at a higher risk of suicide now.”

#### The Importance of Medication Adherence

Provider 1 shared: “If it’s a new medication… a lot of our participants will just stop taking medication.” Respondents described accompanying risks of psychosis (e.g., command hallucinations, paranoia), erratic behaviors, and increased substance use, which increases risk of aggressive behaviors. Provider 7, “Especially schizophrenia, that’s a big one, especially when they’re not taking their medications as prescribed. And then a lot of substance use, mainly substance use diagnoses are what cause negative interactions with first responders than psychotic disorders.” Provider 9, “You might have people experiencing auditory hallucinations, command hallucinations… paranoid delusions, and might be thinking other guests are acting against them … Sometimes it’s just somebody [else] who is responding to internal stimuli, and other guests get irritated and angry at people doing this, and they’ll instigate conflict because of it.”

### Crisis Prevention

Respondents were asked about clinical and social-service strategies to reduce behavioral crises.

#### Prevention and Interventions

All respondents reported continuous involvement in organizational crisis prevention training, including Mental Health First Aid (Hadlaczky et al., 2014), Crisis Prevention Institute (Weittenhiller, 2009), and Handle with Care (Casid and Casid, 2012). And their importance to de-escalate crises. Provider 22 shared their experience: “[We use the] Handle with Care model for de-escalation… it’s really about respecting the person, normalizing their response.” Respondents shared many examples in which crisis prevention was used to “normalize” client experiences to prevent escalation. “Normalizing” in this context refers to reducing further pathologizing and criminalizing the client’s natural response irrespective of mental health etiology and symptomatology. Normalizing via verbal de-escalation was reported useful to stabilize escalated clients. Provider 23 shared additional insight into the de-escalation process: “Someone disturbed with internal stimuli or certain things around them; we’ve got some [grounding] techniques to get them to the focus on their senses*.*” These techniques seek to reorient escalated individuals ’ awareness to their senses through listening, seeing, and touching (Najavits, 2002).

#### Other Prevention Measures and Social Determinants

Respondents highlighted chronic homelessness as major determinant. Provider 17 highlighted their organization’s efforts to promote stable housing, noting that “Housing is obviously the biggest preventative action that we take.” Provider 6 shared: “Part of the prevention is covering the basics of health, connecting people to primary care, getting referrals to behavioral health care.”

### Decision-Making Process: Calling 911

Respondents reported an average of 2.6 calls to 911 during a given month prior to COVID-19. Rationale for calls included safety concerns related to aggressive behavior, and liability concerns embedded in organization policy.

#### Aggressive Behaviors and Safety Concerns

Providers witnessed property destruction. Provider 12 recalled: “We’ve had clients actually throw bricks at our office window when they’re in a mental health crisis. And we’ve also had a client attack another client in front of a staff member, he physically choked her …[I]t was a staff risk, safety concern. And then when police also don’t come right away, clients tend to get more hostile and aggressive.”

### Organizational Policy

Many organizations have explicit policies that govern decisions to call 911. Provider 5 emphasized, “Our policy is not threatening, no violence, no physical acts. So, once we see that happen, they are discharged.” Provider 20 shared their policy, “Call 911 and ask questions later. Providers that do call 911 follow strict protocols to assure safety for all parties.” Decisions to call 911 are highly influenced by staff members’ inability to restrain clients during crises safely and with immunity. Provider 18 describes this decision-making process as, “If we were going to place ourselves in a position where we’re physically manipulating the member [to reach the] hospital, then we’re posing a lot of risks to ourselves by doing that just by the nature of physically restraining or holding someone against their will.” Liability concerns play a key role. Provider 18, “[There are legal challenges] if something happens in the course of restraining or manipulating them. What are we liable? For example… what if the member is suicidal and decides to jump out of the car [on the way to the hospital]. Because we were facilitating that, we’re liable because we put them in a situation.” In similar fashion, Provider 16 shared, “We do rely on police or EMTs in some capacity just for safe transport if a member is unwilling.” Provider 2, “We have to call the first responders.” Within this context, calling first-response is revealed as a means of voluntary or involuntary transportation to avoid liability from clients that might require hospitalization.

### Decision-Making: Not Calling 911

Respondents also discussed why they *may not* call 911 during behavioral crises. These included threats to client and staff safety arising from such calls, risks of incarceration or violence, and concerns about likely effectiveness of first-response. Providers also discussed alternative de-escalation strategies, including redirecting clients to meet with their existing providers before calling 911.

#### Scarcity of CIT Officers, and Fear of Client Harm

Scarcity of CIT-trained officers provided another common reason for not calling 911. Every respondent expressed positive experiences with CIT-trained officers. Many underscored that there are not enough CIT-trained officers to meet community needs or to guarantee that a CIT officer would be sent if one were requested. Provider 22 shared that officers sometimes escalate crises, which “Every time when I have to call the police, I second guess…The situation could be escalating, but I’m still there in my mind figuring out do I really need to call? Can I just wait it out? [There are] only a handful of CIT officers in the two districts in my area.” Provider 15 similarly shared: “Sometimes the police will show up, and they’re just simply too aggressive.” However, “if you can request a CIT officer, then they’re more trained about de-escalating a mental health crisis and that’s great, but there’s hardly any.” Fear that a client in crisis might be injured, incarcerated, or re-traumatized provided another reason for not calling 911. Provider 4 shared: “I never want to see a kid end up in handcuffs that needs to be in the hospital.” Overall, providers referred to calling 911 as a “last resort.” As Director 3 elaborated: “We really try not to call 911 unless we really have to. It’s generally traumatic for staff. It’s traumatic for the participant, and could escalate things. [Our alternative approach includes] pulling in a staff member who may have a better or longer relationship with someone. So using [our] human capital is always helpful.”

### The Experience of 911 Response

Respondents were asked about the aftermath of behavioral crises, including follow-up protocols and services.

#### Client Information Sharing During Crisis

Providers report attempting to give first responders critical information, including clients’ medications, triggers, and preferred hospital. Providers reported trying to accompany clients to the hospital and giving pertinent client information to hospital staff. Provider 8 shared that providers go to the hospital “if possible, and if you can’t, call the [hospital’s] social worker ahead of time.” Provider 6 similarly shared: “We do ask what hospitals they’re taking the person to. If it is involuntary, you can write [in the petition for involuntary admission that] you want to be contacted. And so we put our contact information. Sometimes we will even ride along to the hospital because the client will want us to… [Often, we] end up getting a call [from the hospital requesting information] or we’ll call, or [we’ll simply] be at the emergency room with that person.” Provider 18 described the importance of Assertive Community Treatment (ACT) before and after hospitalization: “[The ACT model spans from] discovering the mental health crisis, to facilitating the hospitalization, [including] emergency services [and] getting [the member] to the hospital, [as well as] following up with the hospital [and the] member while they’re at the hospital, [in addition to] making sure the medications make sense, or that everybody has all the information they need to make the decisions they need to make when they’re getting discharged, [including] ‘Where are they going?’ ‘What’s the plan to continue to maintain that stability after getting discharged from the hospital?’ ACT does all of that.” Many respondents noted that first responders were required to take the client to the nearest hospital, and that there was some tension when providers didn’t believe the nearest hospital provided the ideal care to their client. Provider 16 stated that she advocates for her clients by suggesting to first responders where to take them, and that she often directs the ambulance to specific hospitals. However, others indicated that an ambulance is required to reach the nearest hospital, which often is undesirable, and paramedics or fire are seldom dispatched.

#### Follow-Up Care

Respondents shared that post-hospitalization follow-up depended on the availability of community providers. Provider 11 described post-hospitalization follow-up as “*so disjointed*.” Provider 7 shared that “community resources… are so lacking” and that when clients are discharged, “It’s like, well, they’re still in crisis. So then we have to call the police again… [who] take them right back to that hospital. It’s just this whole cycle… we can’t do anything about this*.*” Several respondents underscored the “revolving door.” Echoing frustrations expressed by Chicago CIT officers (Wood et al., 2021), Provider 11 described the process as: “Someone gets into treatment, they’re released a few days later, they’re still symptomatic, and then they end up behaving in the same way.”

### Provider Recommendations

Respondents were asked how they would spend a “modest grant” to improve their agency’s ability to prevent or address behavioral crises.

#### Organizational and Staff Changes

Many respondents highlighted the need to train existing staff in mental health and to hire additional clinicians. For example, Director 2 underscored the value of greater staff training to promote empathy and restraint in response to various forms of behavioral dysregulation that are uncomfortable to witness, but do not pose a safety threat or require 911 response: “I really would like to spend money on having my entire staff [well trained] on psychiatric issues… they don’t get much training on what decompensation looks like… I would like a lot more training in that area because [our staff] would be more empathetic.” Provider 10 underscored the need to hire additional professionals, and shared: “I believe that the best use of the money would be to have somebody, like a psychiatrist… with the ability to [immediately] prescribe medication… and help them get housing.” Provider 11 highlighted the importance of a diverse workforce. “We’d want to make sure that it’s people who are familiar with the community areas across the city. For example, in a primarily black neighborhood, we don’t want all white clinicians.”

#### Reconstructing the Social Safety Net

Respondents emphasized the importance for all first responders to receive CIT training. Many, however, noted a subtle tradeoff: Self-selection of specifically motivated officers into CIT improves first-response when these officers are available. Yet voluntary training exacerbates the shortage of CIT officers when crises arise. Respondents further underscored that police officers are often put into positions beyond the proper scope of their role. Provider 25 noted that officers “shouldn’t be the safety net. They’re not trained to do this…. They can only do so much.”

#### Streamlining helpline and 911 services

Many respondents cited the NAMI helpline as a useful resource, especially when coordinated with the 911 system. Provider 11 suggested that “having someone from [NAMI’s] helpline or having a team [like NAMI’s] merged within 911” could improve crisis response. Provider 24 suggested that this streamlined system could “deflect calls if [NAMI helpline workers] were sitting in [Chicago’s 911 call center] where they wouldn’t have to reach past a call taker.”

#### Integration of Care Coordination & Alternatives to Police Response

Providers also highlighted the need for better integration of first responders, behavioral health providers, and hospital staff. This includes better coordination of outpatient and inpatient services as well as communication streams to focus more explicitly on community-based prevention interventions. Provider 11 suggested “making sure that all hospital systems in the city have a follow-up process” and that there is “communication when an officer fills out an involuntary petition, making sure someone reaches out.” Provider 25 highlighted the need for non-police community-centered responses, and suggested a “robust mobile crisis response system that isn’t so dependent on insurance… where people can go and feel safe, regardless of whether they’re coming in via an officer or not.” Provider 22 further noted: “Most of the people we service had had negative, if not traumatic, experiences with first responders, especially the police… they will see a person in uniform and that brings them back to a bad experience.” Provider 23 went on to suggest: “Ideally, it would not be uniformed police officers…just seeing an officer show up in uniform, badge, guns, can be triggering and traumatizing.” Provider 21 added: “it’s not a crime to have a mental health crisis, you know?”

#### Housing

Clinicians underscored housing as the crucial first step to an individual gaining stability and avoiding hospitalization. Provider 25 shared, “We cannot work on safety, we cannot work on getting into therapy, we cannot work on medication management, if people are so unstably housed.”

#### Community-Based Services that Address SUD and Co-occurring Disorders

Provider 14 shared: “Substance use rules are pretty antiquated in that the services have to be delivered within four walls, which means basically group or individual therapy, and I think people need so much more than that.” Community-based services, including ACT and Community Support Teams (CST), were noted as valuable additions.

## Discussion

We sought in these interviews to explore when and why providers seek assistance from first responders and how these responses unfold, as well as what outcomes follow first response and recommendations providers might offer for improvement. Our findings describe the lifecycle of behavioral health crisis response across a diverse field of respondents. Respondents revealed that the decision to call 911 occurred 2–3 times monthly and was primarily related to the need to involuntary petition individuals for transfer to a hospital environment. Respondents desired, but did not always receive, CIT trained officers to respond to the 911 call as means of needed restraint and transportation in the face of safety and liability concerns. Outcomes of calls were often positive, but sometimes undesirable, and deemed inappropriate or inadequate with respect to who arrived, or did not arrive, on the scene, as well as where individuals were transported. These issues were related to either the lack of available CIT officers or issues in the flow of information through 911 dispatchers. Respondents’ insights and recommendations for 911 dispatchers, or a “crisis response system” beyond CIT trained officers, as consolidated below, may help shape protocols and policy efforts to improve interactions between first responders and individuals experiencing behavioral crises.

In response to 911 calls, providers may receive police, fire, or paramedics. Providers reported having little choice in who responds to the call. Most often, it is non-CIT trained officers. Respondents indicated that CIT trained officers are the most desirable, as previously reported (Comartin et al., 2019; McKenna et al., 2015), but are still not ideal responders, noting that the presence of uniformed officers may be triggering and inherently escalating. Paramedics and fire were seldom dispatched or unwilling to respond. Chicago is now deploying specific service models to provide alternative response that avoids, minimizes, or complements dispatch of armed officers to behavioral-crisis calls. Under the Crisis Assistance Response and Engagement (CARE) model, multi-disciplinary response teams, unarmed responders, and social-service co-responders are dispatched alongside police to identified high-risk 911 calls. CARE teams seek to coordinate follow-up services (Pollack & Watson, 2020). Within the first six months of pilot implementation, CARE teams reported zero arrests and zero use-of-force incidents in responding to behavioral-health-related calls (Masterson et al., 2022).

Law enforcement personnel may correspondingly assist social service responders by maintaining safety and logistical help. A role for transportation falls on police due to qualified immunity, which protects law enforcement officials from unjustified lawsuits (Warren & Supreme Court of the United States, 1966). In other words, providers and their organizations prefer police responses over potential lawsuits, even if it might result in dangerous outcomes to individuals living with mental illness. Legislation that extends qualified immunity to non-police officials would be required to relieve law enforcement from the transportation role. If this occurs, providers indicated that law enforcement will remain desirable for situations that demand protection during severe crises.

First responders are unable to address broader social and health determinants underlying the emergence of behavioral health crises. Indeed, prior studies involving interviews with police officers indicate that when individuals are “going off meds” this frequently results in mental health related calls (Wood et al., 2021). Our respondents emphasized the importance of medication non-adherence, which heightens risks of potential decompensation or relapse that might escalate to aggression or behavioral crisis. Interventions and research to increase medication adherence are thus specifically important for this context (Robertson et al., 2014). Previous research also underscores the role of housing and other services to meet basic needs in ameliorating health inequities. The role of housing in addressing health inequities is especially pronounced for Medicaid recipients living with severe mental illness and experiencing homelessness (Fenelon et al., 2017; Martone & Martone, 2014; Mathis & Mathis, 2020). Additionally, our respondents noted that substance use disorder might exacerbate adverse effects or pose safety concerns, which is consistent with findings from the current literature (Bride et al., 2015; O’Brien et al., 2021; Weiss et al., 2017). Notably, several of the agencies in our study utilize Integrated Dual Diagnosis Treatment (IDDT) for individuals with co-occurring substance use disorders (Kikkert et al., 2018).

Our respondents lamented the “revolving door,” which describes the process by which individuals in crisis quickly cycle back through the emergency care system, receiving minimal or poor follow-up services until the next 911 call is made. Providers shared that this cycle is accelerated by the low supply of affordable, accessible, and high-quality mental health services for individuals with complex needs. Many hospitals, facing payment models implicitly incentivize high bed turnover, may also lack proper incentives to address this concern (Heggestad & Heggestad, 2001). Integrated services, such as those found within therapeutic communities (Bunt et al., 2008), may provide superior care as an option to bypass the revolving door. Improved follow-up—what might be termed “second response”—is thus essential (Pollack & Watson, 2020).

Our work includes important limitations. Data from separate interview guides were coded together in the absence of inter rate reliability metrics. Our sample were mostly white identifying Males and Females within large provider organizations. Our ongoing work explores views of first responders, families, and service consumers. Moreover, our Chicago findings may not generalize across all settings and localities. We also do not provide granular discussion of 911 telecommunication professionals and others with distinctive roles in the emergency response system.

Our findings underscore both the necessity and limitations of well-managed emergency first-response. While respondents stated that crisis response has improved over time, increased communication to facilitate trust in outcomes of first response is needed. Additionally, response only address immediate encounter, and not prevention or follow-up. Respondents shared many frustrations also expressed by first responders—including the “revolving door,” whereby individuals in crisis cycle in and out of hospitals without access to long-term quality mental health care. Respondents particularly hoped to see greater attention and resources provided to social determinants, especially housing. Without systematic efforts to address social determinants, this revolving door cannot close.

## References

[CR1] Bonfine N, Wilson AB, Munetz MR (2020). Meeting the needs of justice-involved people with serious mental illness within community behavioral health systems. Psychiatric Services.

[CR2] Bride BE, Choi YJ, Olin IW, Roman PM (2015). Patient violence towards counselors in substance use disorder treatment programs: Prevalence, predictors, and responses. Journal of Substance Abuse Treatment.

[CR3] Bunt GC, Muehlbach B (2008). The Therapeutic Community: International perspective. Substance Abuse.

[CR4] Casid JH (2012). Handle with care. TDR/The Drama Review.

[CR5] Comartin EB, Swanson L, Kubiak S (2019). Mental health crisis location and police transportation decisions: The impact of crisis intervention team training on crisis center utilization. Journal of Contemporary Criminal Justice.

[CR6] Compton, M. T., Bakeman, R., Broussard, B., ... & Watson, A. C. (2014). The police-based crisis intervention team (CIT) model: II. Effects on level of force and resolution, referral, and arrest. *Psychiatric Services,**65*(4), 523–529. 10.1176/appi.ps.201300108. 10.1176/appi.ps.201300108 24382643

[CR7] Compton MT, Broussard B, Hankerson-Dyson D, Krishan S, Stewart T, Oliva JR, Watson AC (2010). System- and policy-level challenges to full implementation of the crisis intervention team (CIT) model. Journal of Police Crisis Negotiations.

[CR8] Cordner, G. (2006). Problem-oriented guides for police. *Problem-Specific Guides Series*. US Department of Justice.

[CR9] Deane M, Steadman H, Borum R, Veysey B, Morrissey J (1999). Emerging partnerships between mental health and law enforcement. Psychiatric Services.

[CR10] Epperson MW, Wolff N, Morgan RD, Fisher WH, Frueh BC, Huening J (2014). Envisioning the next generation of behavioral health and criminal justice interventions. International Journal of Law and Psychiatry.

[CR11] Fenelon, A., Mayne, P., Simon, A. E., ... & Steffen, B. (2017). Housing assistance programs and adult health in the United States. *American Journal of Public Health*, *107*(4), 571–578 10.2105/AJPH.2016.303649 PMC5343706 28207335

[CR12] Fuller D, Lamb R, Biasotti M, Snook J (2015). Overlooked in the undercounted: The role of mental illness in fatal law enforcement encounters.

[CR13] Hadlaczky G, Hökby S, Mkrtchian A, Carli V, Wasserman D (2014). Mental Health First Aid is an effective public health intervention for improving knowledge, attitudes, and behaviour: A meta-analysis. International Review of Psychiatry.

[CR14] Heggestad T (2001). Operating conditions of psychiatric hospitals and early readmission—Effects of high patient turnover. Acta Psychiatrica Scandinavica.

[CR15] Hoch J, Hartford K, Heslop L, Stitt L (2009). Mental illness and police interactions in a mid-sized Canadian City: what the data do and do not say. Canadian Journal of Community Mental Health.

[CR16] Kikkert M, Goudriaan A, de Waal M, Peen J, Dekker J (2018). Effectiveness of Integrated Dual Diagnosis Treatment (IDDT) in severe mental illness outpatients with a co-occurring substance use disorder. Journal of Substance Abuse Treatment.

[CR17] Livingston JD (2016). Contact between police and people with mental disorders: A review of rates. Psychiatric Services.

[CR18] Magee LA, Fortenberry JD, Rosenman M, Aalsma MC, Gharbi S, Wiehe SE (2021). Two-year prevalence rates of mental health and substance use disorder diagnoses among repeat arrestees. Health Justice.

[CR19] Martone K (2014). The impact of failed housing policy on the public behavioral health system. Psychiatric Services.

[CR20] Masterson, M. (2022). No arrests , use of force reported in first months of Chicago’s 911 co-responder pilot. *WTTW*. Retrieved March 4 from https://news.wttw.com/2022/03/04/no-arrests-use-force-reported-first-months-chicago-s-911-co-responder-pilot

[CR21] Mathis J (2020). Housing is mental health care: A call for Medicaid demonstration waivers covering housing. Psychiatric Services.

[CR22] McKenna B, Furness T, Oakes J, Brown S (2015). Police and mental health clinician partnership in response to mental health crisis: A qualitative study. International Journal of Mental Health Nursing.

[CR23] Najavits L (2002). Seeking safety: A treatment manual for PTSD and substance abuse.

[CR24] O’Brien P, Henke RM, Schaefer MB, Lin J, Creedon TB (2021). Adverse events among adult Medicaid enrollees with opioid use disorder and co-occurring substance use disorders. Drug and Alcohol Dependence.

[CR25] Padgett DK (2016). Qualitative methods in social work research.

[CR26] Pollack HA, Watson AC (2020). From crisis to care: Improved second response to mental health crises. Milbank Quarterly Opinion.

[CR28] Robertson AG, Swanson JW, Van Dorn RA, Swartz MS (2014). Economic grand rounds: treatment participation and medication adherence: Effects on criminal justice costs of persons with mental illness. Psychiatric Services.

[CR29] Teherani A, Martimianakis T, Stenfors-Hayes T, Wadhwa A, Varpio L (2015). Choosing a qualitative research approach. Journal of Graduate Medical Education.

[CR30] Tentner AR, Spellman A, Paulson A, Day C, Sadler T, Coffman R, Pollack HA (2019). Identifying Chicago’s high users of police-involved emergency services. The American Journal of Public Health.

[CR31] Teplin L, Pruett N (1992). Police as streetcorner psychiatrist: Managing the mentally ill. International Journal of Law and Psychiatry.

[CR32] Teplin LA (1983). The criminalization of the mentally-Ill—Speculation in search of data. Psychological Bulletin.

[CR100] Warren, E., & Supreme Court of the United States. (1966). U.S. Reports: Pierson V. Ray, 386 U.S. 547. Periodical. https://www.loc.gov/item/usrep386547/

[CR33] Watson AC, Compton MT (2019). What research on crisis intervention teams tells us and what we need to ask. Journal of the American Academy of Psychiatry and the Law.

[CR34] Watson AC, Compton MT, Draine JN (2017). The crisis intervention team (CIT) model: An evidence-based policing practice?. Behavioral Sciences & the Law.

[CR35] Watson AC, Owens LK, Wood J, Compton MT (2021). The impact of crisis intervention team response, dispatch coding, and location on the outcomes of police encounters with individuals with mental illnesses in Chicago. Policing (Oxford).

[CR36] Watson AC, Pope LG, Compton MT (2021). Police reform from the perspective of mental health services and professionals: Our role in social change. Psychiatric Services.

[CR37] Weiss NH, Connolly KM, Gratz KL, Tull MT (2017). The role of impulsivity dimensions in the relation between probable posttraumatic stress disorder and aggressive behavior among substance users. Journal of Dual Diagnosis.

[CR38] Weittenhiller, J. (2009). *De-escalation and management of students with emotional behavioral disabilities by using techniques from the crisis prevention institute and the response goal-oriented intervention model*.

[CR39] Wood JD, Watson AC, Barber C (2021). What can we expect of police in the face of deficient mental health systems? Qualitative insights from Chicago police officers. Journal of Psychiatric and Mental Health Nursing.

